# Efficacy and safety comparison between 1927 nm thulium laser and 2940 nm Er:YAG laser in the treatment of facial atrophic acne scarring: a prospective, simultaneous spilt-face clinical trial

**DOI:** 10.1007/s10103-021-03465-0

**Published:** 2021-11-26

**Authors:** Kune Lu, Suiqing Cai

**Affiliations:** Department of Dermatology, The Second Affiliated Hospital of Zhejiang University School of Medicine, Hangzhou, China

**Keywords:** Acne scar, 1927 nm thulium laser, 2940 nm Er:YAG laser, Fractional laser

## Abstract

Acne scarring is a common disfiguring complication of acne, and fractional lasers are widely applied in improving it. This study is to compare the efficacy and safety of fractional non-ablative 1927 nm thulium laser (FTL) and fractional ablative 2940 nm Er:YAG laser (FEL) in the treatment of acne scarring. Subjects with moderate or severe atrophic facial acne scarring received 3 sessions of FTL on the left side of face and FEL on the right side of face at an average interval of 4–6 weeks. Major assessments included Goodman&Baron quantitative global scarring grading system (GBS), self-rated improvement and satisfaction score. Twenty-seven subjects completed the study; for FTL side, average GBS decreased from 11.15 ± 5.04 at baseline to 7.07 ± 4.87 with an improvement percent of 36.54%; for FEL side, average GBS decreased from 10.81 ± 4.46 to 7.00 ± 4.07 with an improvement percent of 35.27%. Adverse effects include transient pain, erythema, edema, and increase of acne. No significant difference was found between two lasers. Both FTL and FEL improved atrophic acne scarring and were well-tolerated. Increase of acne during laser treatment may have a negative impact on efficacy. Trial registration number was NCT04813419 and date of registration was 19th, March, 2021, retrospectively registered.

## Introduction

Acne vulgaris is chronic and recalcitrant inflammation of pilosebaceous unit that has a high incidence rate in adolescence and even adults. Acne scarring is not an uncommon cosmetic complication which may cause physical and psychological pressure and impair the life quality of patients [1]. It can be divided into two types according to morphology: atrophic and hypertrophic acne scarring. Atrophic acne scarring can be subclassified into boxcar, icepick, and rolling scarring due to morphological features [2]. Although a great variety of modalities to treat atrophic acne scarring such as chemical peeling, lasers and light, micro-needling, and radiofrequency have emerged, fractional lasers (FLs) have come out on top [3]. Unlike resurfacing lasers, FLs create three-dimensional, evenly distributed “microscopic thermal zones (MTZs)” on the treating area, which only covers about 3–40% of the skin and leaving the surrounding tissue undamaged, serving as “cell reservoir.” Then, the MTZs can be rapidly replaced by keratinocytes in “cell reservoir” within the first 24 h and by new collagen within 3–6 months; thus atrophic acne scarring was improved [4]. FLs can be categorized into fractional ablative lasers (FALs) and fractional non-ablative lasers (FNALs) depending on wavelength.

With a wavelength of 2940 nm, FEL could be highly absorbed by water-containing tissues of skin and cause superficial epidermis ablation and collagen induction [4]. But thermal damage is limited to about 20–50 μm [5]. It has been reported to be effective in improving acne scars through upregulating transcription of transforming growth factor (TGF) βs, collagenases, and tissue inhibitor of metalloproteinase (TIMP) [6, 7]. FTL has a moderate affinity for water content tissue. Thus, rather than causing epidermis turnover, it keeps the epidermis intact. But it can penetrate deep into 200–300 μm and stimulate modest collagen regeneration [8]. An animal study revealed that after FTL treatment, epidermal necrosis, and collagen denaturation at the upper dermis were induced [9].

Prior studies had shown that both FALs and FNALs were effective in treating acne scarring and the FALs were more effective, while the FNALs had fewer side effects such as hyperpigmentation [10, 11]. However, in our clinic, we have observed significant effect and high satisfaction rate of FTL in improving atrophic acne scarring. Since there was only one clinical trial reported the efficacy and safety of FTL in Asian and no study have made a comparison between FTL and FEL, we designed this prospective and simultaneous spilt-face trial, hoping to provide a new available modality for patients who are unable to tolerable or are reluctant to ablative lasers.

## Materials and methods

### Study design and patient characteristics

This was a prospective, simultaneous spilt-face clinical trial. Patients with facial atrophic acne scarring received 3 sessions of FTL and FEL respectively at an average interval of 4–6 weeks. The study had been carried out in accordance with Declaration of Helsinki, and the protocol was approved by the ethic committee of our hospital. Written informed consent was obtained from each subject before enrollment. Inclusion criteria were adults (at least 18 years old) and similar atrophic acne scarring on both sides of face; exclusion criteria includes the following: (1) There was infection in the treatment site; (2) acne vulgaris was not controlled; (3) had a propensity for keloid forming; (4) received oral isotretinoin or laser treatment in the past 3 months; (5) received chemical peeling 1 month before the study; and (6) pregnancy or breast-feeding.

### Treatment

Topical anesthetic was administered prior to laser therapy to reduce the pain. We treated the left side with FTL (Lavieen, Korea). Parameters were set as stamp mode, a spot size of 5*5 mm, a pulse time of 1000 μs, an energy of 10 mJ/shot, and pulse stacking varied from 1 to 2. We treated right face with FEL (MCL 31 Asclepion Laser Technologies, Germany), N25 mode, a spot size of 9*9 mm, and a pulse time of 300 μs. Energy intensity was adjusted from 12 to 27 J/cm^2^. Immediately after laser treatment, sterile cooling pad was applied to face for about 20 min. Then, patients were required to apply chlortetracycline ointment twice a day to prevent infection in the first 3 days. Moreover, avoidance of water and sun exposure before the crusts falling off were suggested. All patients received 3 treatment sessions totally at an average interval of 4 to 6 weeks. Follow-up was carried out for 12 weeks after the final treatment. Standardized digital photographs were taken by VISIA facial skin imaging analyzer (Canfield, USA) at baseline (T0), the moment before the third session (T1), and 12 weeks after the final treatment (T2).

### Outcome assessments

Both objective and subjective efficacy evaluation were based on comparing standardized digital photographs taken before and after laser treatments. Objective evaluation was the Goodman&Baron quantitative global scarring grading system (GBS) evaluated by a blinded dermatologist. The criteria are listed in Table 1 [12]. Subjective evaluation contained acne scar improvement and satisfaction score rated by patients. Self-rated acne scar improvement ranged from 0 to 4 (0, no improvement; 1, 1–25% improvement; 2, 26–50% improvement; 3, 51–75% improvement; and 4, 76–100% improvement). Satisfaction score ranged from 0 to 10, 0 meant not satisfied at all and 10 meant very satisfied. Adverse reactions such as pain, erythema, edema, hyperpigmentation, and hypopigmentation were recorded during the whole research time. Pain score was evaluated immediately after each laser treatment with visual analogue scale (VAS) 0 to 10, 0 meant no pain, while 10 meant extremely painful.

**Table 1:** Goodman&Baron quantitative global scarring grading system (GBS)

Type	Number of lesions 1 (1–10) 2 (11–20) 3 (> 20)
Milder scarring (1 point each) Macular erythematous or pigmented Mildly atrophic dish-like	
(B) Moderate scarring (2 points each) Moderately atrophic dish-like Punched out with shallow bases, small scarring(< 5 mm) Shallow but broad atrophic areas	
(C) Severe scarring (3 points each) Punched out with deep but normal bases, small scarring (< 5 mm) Punched out with deep abnormal bases, small scarring (< 5 mm) Linear or troughed dermal scarring Deep, broad atrophic areas	

### Statistical analysis

All data analyses were performed using SPSS software (version 25.0, SPSS Inc, Chicago, IL), and statistical significance was *P* < 0.05. McNemar’s test was utilized for the evaluation of differences between categorical variables of the two dependent groups. Paired *T*-test and Wilcoxon sign-rank test were used for the evaluation of differences in paired continuous data, while independent *T*-test and Mann–Whitney U-test were used for grouped continuous data with normal and non-normal distributions, respectively.

## Results

### Baseline characters

Thirty patients with moderate to severe atrophic facial acne scarring were enrolled in the study, and 27 of them completed the research. One female patient quitted after one laser session because of increasing acne vulgaris, and the other two male patients quitted after one laser session because of personal reasons. Clinicodemographic information of participants is given in Table 2.

**Table 2:** Baseline demographic and clinical features of participants

Characteristic	Value
Moderate to severe ratio, no Female to male ratio, no	10:17 11:16
Age (range), y	24–32
Duration of acne scar, median(interquartile range),y	7 (1–12)
Fitzpatrick skin type, no. (%)	
III IV V	15 (55.6) 9 (33.3) 3 (11.1)
Predominant scar type, no. (%)	
Rolling Boxcar Icepick Mixed	6 (22.2) 12 (44.4) 7 (25.9) 2 (7.4)
GBS prior to laser treatment	
FEL (median ± SD) FTL (median ± SD) *P* value	10.81 ± 4.46 11.15 ± 5.04 > 0.05

*No.* number, *y* years, *SD* standard deviation, *FTL* fractional 1927 nm thulium laser, *FEL* fractional 2940 nm Er:YAG laser

### Objective evaluation

**Fig. 1:**
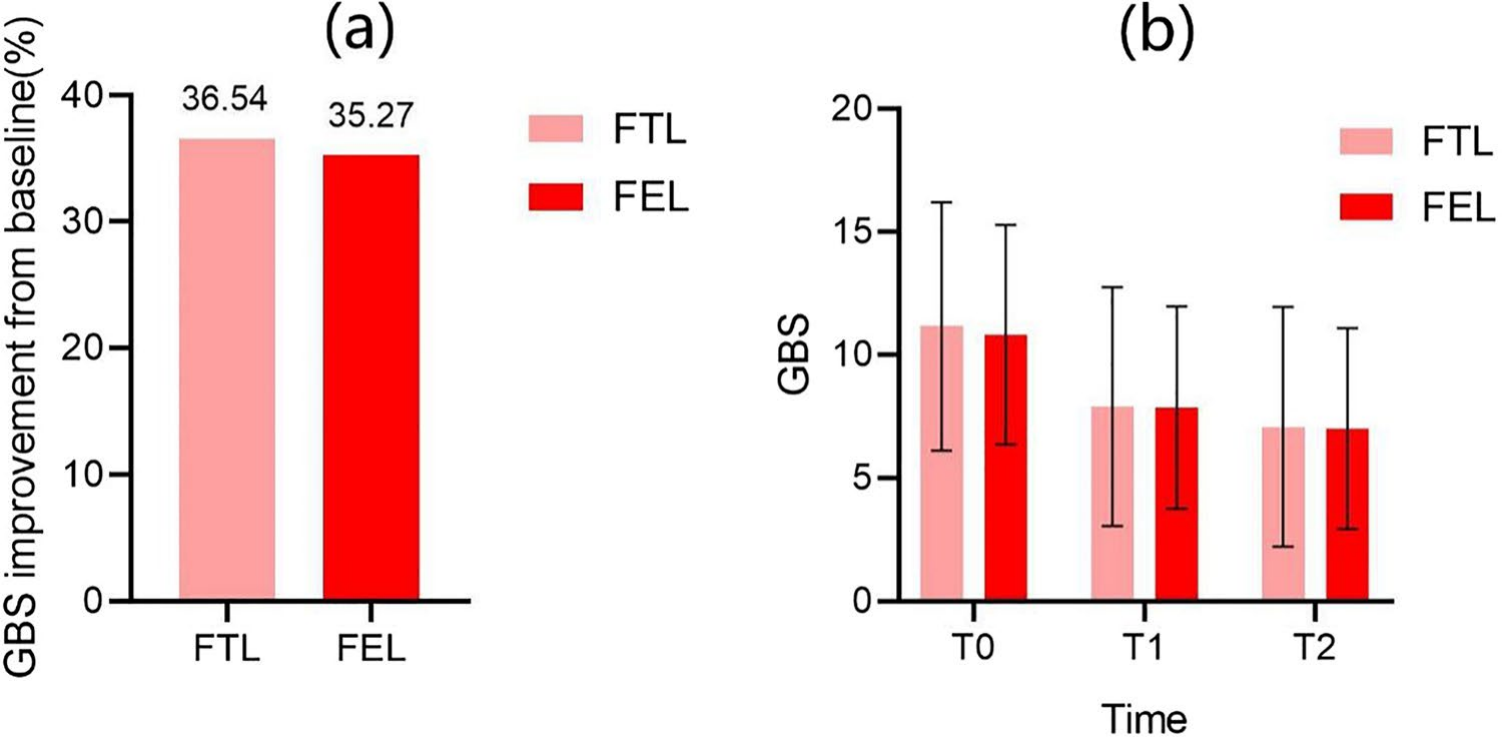
**a** GBS improvement percent from baseline to T2, *P* > 0.05; **b** GBS change from T0 to T1 and T2. FTL: 11.15 ± 5.04 (T0), 7.89 ± 4.85 (T1), 7.07 ± 4.87 (T2); FEL: 10.81 ± 4.46 (T0), 7.85 ± 4.11 (T1), 7.00 ± 4.07 (T2). FTL, fractional 1927 nm thulium laser; FEL, fractional 2940 nm Er:YAG laser

After 3 sessions, the mean GBS of FTL side decreased from 11.15 ± 5.04 at T0 to 7.07 ± 4.87 at T2 (*P* < 0.001) and improved by 36.54%. The mean GBS of FEL side decreased from 10.81 ± 4.46 at T0 to 7.00 ± 4.07 at T2 (*P* < 0.001) and improved by 35.27%. However, there was no statistical difference between the two lasers, *P* > 0.05 (Fig. 1).

### Subjective evaluation

As for patient evaluation, for FTL treatment, average satisfaction score was 6.41 ± 2.42, and average self-rated improvement was 1.89 ± 0.97; for FEL laser treatment, patient satisfaction score was 6.74 ± 2.07, and self-rated improvement was 1.93 ± 0.87. No statistical significance was found between the two lasers.

**Fig. 2:**
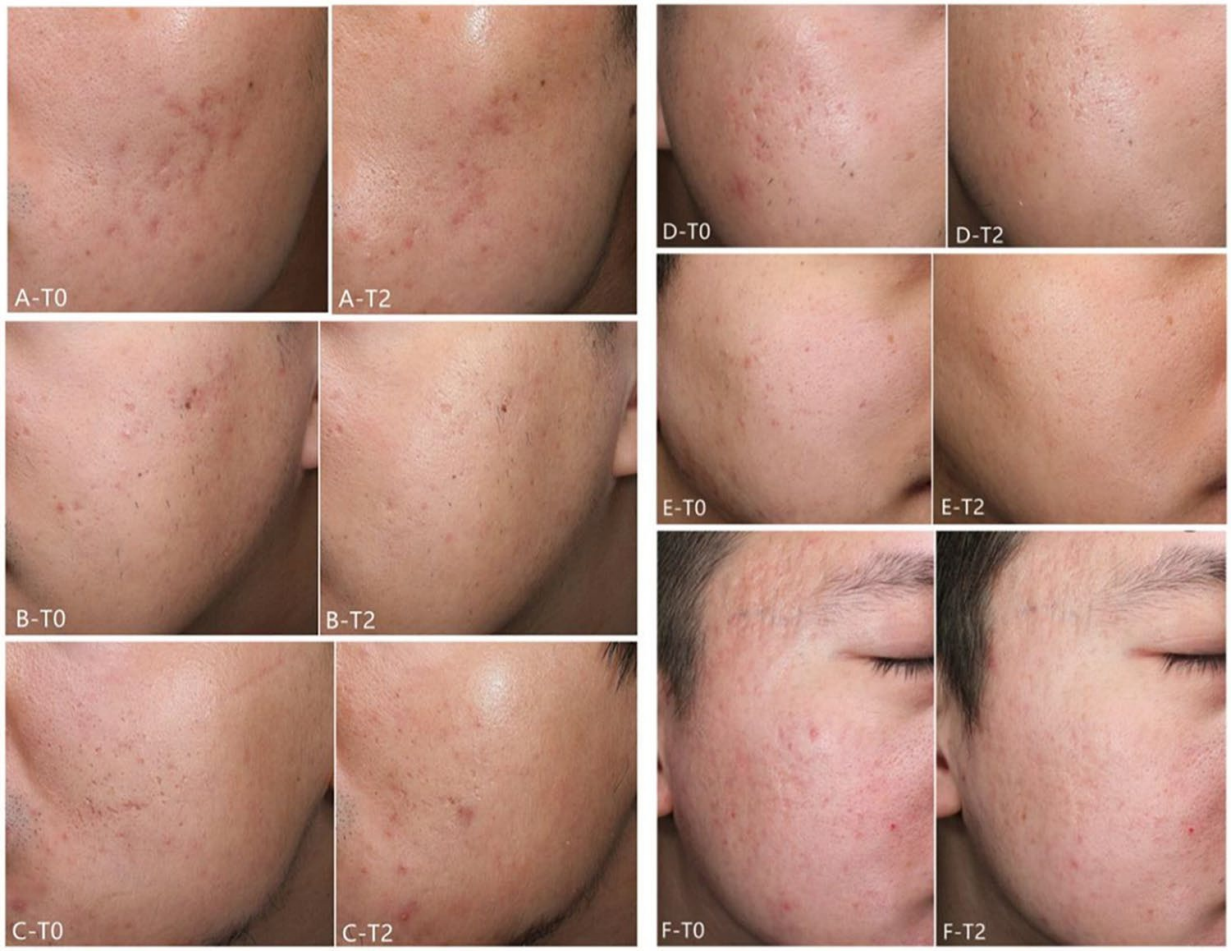
A, B, and C were three patients treated with FTL on left side; D, E, and F were three patients treated with FEL on right side. T0 represented baseline and T2 represented 12 weeks after the final treatment. Remarkable improvements can be seen on pictures

Representative photographs illustrating acne scarring improvement from T0 to T2 are shown in Fig. 2.

**Fig. 3:**
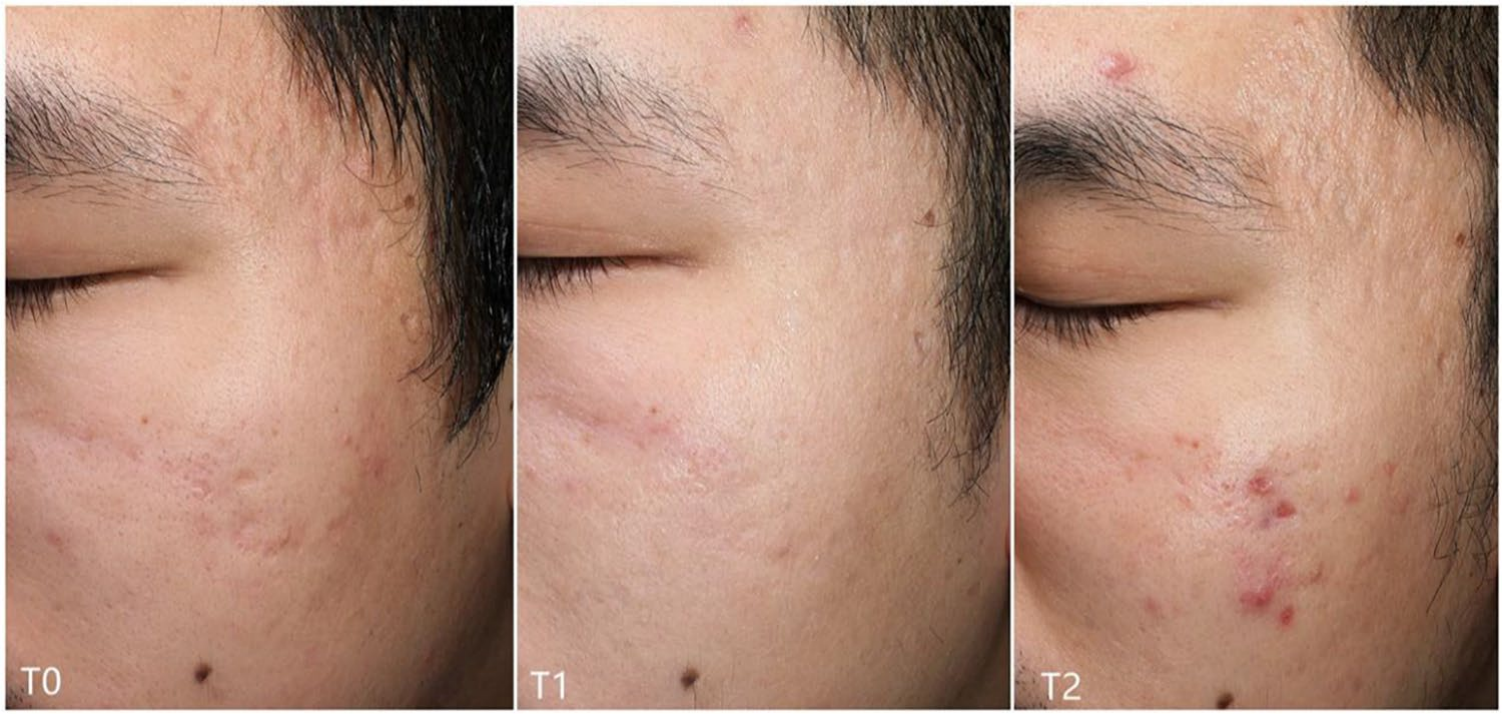
Consecutive pictures showing the acne scarring of T2 were worse than that of T1

Interestingly, we observed that the GBS of T2 was higher than that of T1 (Fig. 3). Thus, further analysis was performed to explore this worsening phenomenon. Relative factors such as age, sex, acne scarring duration, and increase of acne vulgaris were taken into analyzation. Univariate analysis showed that both the increase of acne and scar type were related to acne scar worsening in the 2940 nm laser side while only increase of acne in the 1927 nm laser side. Then, we took increase of acne and scar type into logistic regression analysis and results showed that increase of acne vulgaris was of statistical significance between the worsening (GBS of T2 > T1) and not worsening (GBS of T2 ≤ T1) groups, *P* < 0.05 (Tables 3, 4, 5, and 6).

**Table 3:** Univariate analysis of 2940 nm laser

		Not worsening (GBS of T2 ≤ T1)	Worsening (GBS of T2 > T1)	*P*
Sex	Male	12 (75%)	4 (25%)	1
	Female	9 (81.8%)	2 (18.2%)	
Acne increase	No	19 (90.5%)	2 (9.5%)	0.011*
	Yes	2 (33.3%)	4 (66.7%)	
Scar type	Rolling	6 (100.0%)	0 (0%)	0.029*
	Boxcar	9 (75%)	3 (25.0%)	
	Icepick	6 (85.7%)	1 (14.3%)	
	Mixed	0	2 (100%)	
Fitzpatrick skin type	III	11 (73.3%)	4 (26.7%)	0.598
	IV	7 (77.8%)	2 (22.2%)	
	V	3 (100%)	0 (0%)	
Scar duration, y	7.31 ± 3.09	7.17 ± 2.14	0.917	
Age, y#	26 (25 ~ 29)	26 (25 ~ 28.25)	0.657	

* means *P* < 0.05; # means non-normal distribution after SK normality test. *y* years, *T1* at the moment before the third laser treatment, *T2* at 12 weeks after the final treatment

**Table 4:** logistic regression analysis of 2940 nm laser

	Odds ratio (95% confidence interval)	*P*
Scar type	2.067 (0.527–8.107)	0.298
Acne increase	12.469 (1.146–135.720)	0.038*
No acne increase	Reference	–
Constant	0.022	0.030

* means *P* < 0.05

**Table 5:** Univariate analysis of 1927 nm laser

		Not worsening (GBS of T2 ≤ T1)	Worsening (GBS of T2 > T1)	*P*
Sex	Male	10 (62.5%)	6 (37.5%)	0.183
	Female	10 (90.9%)	1 (9.1%)	
Acne increase	No	19 (90.5%)	2 (9.5%)	0.001*
	Yes	1 (16.7%)	5 (83.3%)	
Scar type	Rolling	4 (66.7%)	2 (33.3%)	0.075
	Boxcar	10 (83.3%)	2 (16.7%)	
	Icepick	6 (85.7%)	1 (14.3%)	
	Mixed	0	2 (100%)	
Fitzpatrick skin type	III	10 (66.7%)	5 (33.3%)	0.462
	IV	7 (77.8%)	2 (22.2%)	
	V	3 (100%)	0 (0%)	
Scar duration, y	7.23 ± 3.06	7.43 ± 2.44	0.875	
Age, y#	26 (25 ~ 28.75)	27 (25 ~ 29)	0.694	

* means *P* < 0.05; # means non-normal distribution after SK normality test. *y* years, *T1* at the moment before the third laser treatment, *T2* at 12 weeks after the final treatment

**Table 6:** logistic regression analysis of 1927 nm laser

	Odds ratio (95% confidence interval)	*P*
Scar type	0.482 (0.088–2.636)	0.400
No acne increase	Reference	0.014*
Acne increase	116.485 (2.643–5134.405)	–
Constant	0.595	0.410

* means *P* < 0.05

In terms of side effects, average treatment-related pain score assessed by patients was 2.74 ± 1.63 on the FTL side and 3.47 ± 2.22 on the FEL side (*P* < 0.05). Other side effects include transient erythema, edema, and increase of acne. No hypopigmentation or hyperpigmentation was observed in both groups (Table 7). Crusts fell off within 5–7 days on the FEL side and 3–5 days on the FTL side.

**Table 7:** Summary of side effects of two lasers

	FTL	FEL
Erythema, days	5.5 (3–7)	5.5 (3.25–7)
Hypopigmentation	None	None
Hyperpigmentation	None	None
Acne increase	6/27 (22.22%)	6/27 (22.22%)
Pain score(VAS)	2.74 ± 1.63	3.47 ± 2.22

*FTL* Fractional 1927 nm thulium laser; *FEL* fractional 2940 nm Er:YAG laser; *VAS* visual analogue scale

## Discussion

In this prospective clinical trial, patients with atrophic acne scarring responded well to FTL and FEL. The acne scar appearance improved about 36.54% (FTL) and 35.27% (FEL) respectively without significant difference between two lasers. Adverse effects included transient erythema, edema, pain, and increase of acne. There was no statistical difference between them in other aspects, except in the pain score, of which FEL was slightly higher than FTL.

FL has been used to treat acne scar for a long time. Our data demonstrated that both FTL and FEL were effective in improving acne scarring, which was consistent with previous studies. Firooz et al. reckoned that FEL is a user-friendly and effective system for skin phototypes III–IV and reported fair to good improvement of all patients according to subjective assessment [7]. Min et al. reported an improvement of 50% on FEL side in one spilt-face trial [6]. A retrospective study reported about 70% of patients acquired 26–50% acne scar improvement after 3 FEL treatments [13]. One Chinese study observed remarkable improvements with ECCA score decreased from 80.23 ± 6.22 to 31.21 ± 3.43 after three sessions of FTL at an interval of 40 days and a follow-up period of 3 months. Biopsies from atrophic acne scar revealed a decrease in the dermal thickness and loss of sebaceous glands [14]. And FL could induce wound healing process and promote collagen remodeling to improve the appearance of acne scars [9, 15]. The accurate molecular mechanisms have not been clarified. However, it has been reported that FEL could upregulate matrix metalloproteinase (MMP)1, MMP13, TGFβ3, and TIMP and downregulate peroxisome proliferator-activated receptor gamma (PPARγ) to increase collagen expression [6].

Prior studies showed that FALs were more effective than FNALs [10, 16]. However, our study did not show significant difference between two lasers. Three possible reasons for non-ablative FTL performing as well as ablative FEL laser are listed below. Firstly, although FTL belongs to non-ablative laser according to wavelength, it has higher affinity for water than traditional FNALs like the 1550 nm laser. Though it cannot cause epidermis vaporization like FALs, histological analysis had shown coagulation necrosis of epidermal cells and relatively intact stratum corneum [9]. Still, it can penetrate deep into papillary dermis [17, 18]. These indicate that FTL is more like semi-ablative laser. Secondly, lasers take effect beyond selective photothermal effect. Two studies comparing ablative 10,600 nm carbon dioxide laser with non-ablative 1064 nm laser yielded conflict result. One trial shown 10,600 nm laser worked better, while the other one shown similar efficacy. The major difference lied in two studies was pulse width of 1064 nm laser. One is millisecond 1064 nm laser, while the other was picosecond 1064 nm laser. When the pulse width is shortened from millisecond to picosecond, lasers produce not only selective photothermal effect but also photomechanical effect. Similarly, aside from selective photothermal effect, FTL had other mechanisms. Histology of atrophic acne scar have revealed that in addition to the decrease of dermal collagen fibers, there was also a significant decrease of hair follicle sebaceous units [14]. FTL can promote pilosebaceous unit regeneration with unclear mechanism [19]. Therefore, we hypothesis that FTL treat acne scarring not only through inducing dermal collagen regeneration, but also increase the thickness of dermis by promoting the proliferation of pilosebaceous units. Thirdly, because the treatment course is insufficient or the follow-up time is short, FEL has not shown or not reach statistical significance, since the improvement of acne scar by laser is cumulative effect and is closely related to the parameter setting. A pilot randomized spilt-face study performed only one session of 10,600 nm laser and 1550 nm laser; no statistical difference was found as well [20]. In our study, the initial application energy of 2940 nm laser treatment side was relatively low, about 12-18 J, and all patients only received three treatment sessions. Moreover, we only followed 12 weeks.

Another interesting finding of our study is that the acne scarring of T2 was worse than T1 in some patients on both sides, which had never been discussed before, to our best knowledge. No statistical difference was found between the two modalities. Increase of acne vulgaris seems to be closely related to the aggravation. It is not difficult to understand because acne scarring was the product of wound healing process from inflammation of acne vulgaris. The increase of matrix metalloproteinases (MMPs), enzymes degrading extracellular matrix, has been detected in acne [21], and the imbalance between MMPs and tissue inhibitors of MMPs results in hypertrophic or atrophic scarring [2]. For our subjects, collagen degradation and destruction due to inflammation exceeded induction after fractional lasers. Furthermore, we speculated that the new collagen was immature and instable with only 3 laser treatments, which accelerated this process. It reminds us the importance of curing acne vulgaris before laser therapies and preventing infection after laser therapies.

There are three main limitations in this study. First of all, this study is a simultaneous spilt-face clinical trial. Although the results show that both lasers are effective, it is not sure whether there exists interaction between two lasers, that is, we are not sure if the same results can be achieved when we use only one laser. Secondly, the sample size of this study is small, and follow-up time is too short to observe the effect of longer time, such as 6 months or even 12 months. Finally, this study did not carry out histopathological and molecular analysis, and the mechanism of two kinds of laser to improve acne scar is insufficient, especially the mechanism of FTL.

## Conclusions

In conclusion, this is the first prospective clinical trial comparing the efficacy and safety of FTL and FEL in treating atrophic acne scarring. Both lasers are effective and well-tolerated. Moreover, concrete settings are provided, hoping to help clinicians make their best decision. Besides, it is better to keep in mind the importance of preventing the relapse of acne vulgaris and infection during laser treatment. More well-designed clinical trials with lager samples and histopathology analysis are needed to explore the mechanism and maximize the effect of FTL.

## References

[1] Halvorsen JA, Stern RS, Dalgard F (2011). Suicidal ideation, mental health problems, and social impairment are increased in adolescents with acne: a population-based study. J Invest Dermatol.

[2] Fabbrocini G, Annunziata MC, D'Arco V (2010). Acne scars: pathogenesis, classification and treatment. Dermatol Res Pract.

[3] Bhargava S, Cunha PR, Lee J (2018). Acne scarring management: systematic review and evaluation of the evidence. Am J Clin Dermatol.

[4] Bogdan Allemann I, Kaufman J (2010). Fractional photothermolysis–an update. Lasers Med Sci.

[5] Jeong JT, Kye YC (2001). Resurfacing of pitted facial acne scars with a long-pulsed Er:YAG laser. Dermatol Surg.

[6] Min S, Park SY, Moon J (2017). Comparison between Er:YAG laser and bipolar radiofrequency combined with infrared diode laser for the treatment of acne scars: differential expression of fibrogenetic biomolecules may be associated with differences in efficacy between ablative and non-ablative laser treatment. Lasers Surg Med.

[7] Firooz A, Rajabi-Estarabadi A, Nassiri-Kashani MH (2016). Treatment of atrophic facial acne scars with fractional Er:YAG laser in skin phototype III-IV: A pilot study. J Cosmet Laser Ther.

[8] Lee SJ, Chung WS, Lee JD (2014). A patient with cupping-related post-inflammatory hyperpigmentation successfully treated with a 1,927 nm thulium fiber fractional laser. J Cosmet Laser Ther.

[9] Kwon IH, Bae Y, Yeo UC (2018). Histologic analyses on the response of the skin to 1,927-nm fractional thulium fiber laser treatment. J Cosmet Laser Ther.

[10] You HJ, Kim DW, Yoon ES (2016). Comparison of four different lasers for acne scars: Resurfacing and fractional lasers. J Plast Reconstr Aesthet Surg.

[11] Elsaie ML, Ibrahim SM, Saudi W (2018). Ablative fractional 10 600 nm carbon dioxide laser versus non-ablative fractional 1540 nm Erbium-glass laser in Egyptian post-acne scar patients. J Lasers Med Sci.

[12] Goodman GJ, Baron JA (2006). Postacne scarring–a quantitative global scarring grading system. J Cosmet Dermatol.

[13] Cenk H, Sarac G (2020) Effectiveness and safety of 2940-nm multifractional Er: YAG laser on acne scars. Dermatol Ther e14270 10.1111/dth.14270 32882085

[14] Lee WJ, Jung HJ, Lim HJ (2013). Serial sections of atrophic acne scars help in the interpretation of microscopic findings and the selection of good therapeutic modalities. J Eur Acad Dermatol Venereol.

[15] Taudorf EH, Danielsen PL, Paulsen IF (2015). Non-ablative fractional laser provides long-term improvement of mature burn scars–a randomized controlled trial with histological assessment. Lasers Surg Med.

[16] Asilian A, Salimi E, Faghihi G (2011). Comparison of Q-Switched 1064-nm Nd: YAG laser and fractional CO2 laser efficacies on improvement of atrophic facial acne scar. J Res Med Sci.

[17] Hong SP, Lee HM, Won CH (2011). A patient with Bowen's disease successfully treated using a 1,927-nm thulium fiber fractional laser. Dermatol Surg.

[18] Miller L, Mishra V, Alsaad S (2014). Clinical evaluation of a non-ablative 1940 nm fractional laser. J Drugs Dermatol.

[19] Cho SB, Goo BL, Zheng Z (2018). Therapeutic efficacy and safety of a 1927-nm fractionated thulium laser on pattern hair loss: an evaluator-blinded, split-scalp study. Lasers Med Sci.

[20] Cho SB, Lee SJ, Cho S (2010). Non-ablative 1550-nm erbium-glass and ablative 10 600-nm carbon dioxide fractional lasers for acne scars: a randomized split-face study with blinded response evaluation. J Eur Acad Dermatol Venereol.

[21] Williams HC, Dellavalle RP, Garner S (2012). Acne vulgaris. Lancet.

